# Supplementary material for: Computational Mechanobiology Model Evaluating Healing of Postoperative Cavities Following Breast-Conserving Surgery
Source: bioRxiv. 2023 Apr 28:2023.04.26.538467. Preprint. [Version 1] doi: 10.1101/2023.04.26.538467 (PMC10168325; doi:10.1101/2023.04.26.538467)
Supplement: Supplement 1 [file media-1.pdf]

Supplementary Material

Parameter	Description	Value	Reference
D_c $[mm^2/hr]$	Cytokine Diffusion Coefficient	0.01208	[1, 2, 3]
$d_{\rho,c}$ $[mm^2/hr]$	Cytokine-Increased Fibroblast Diffusivity	6.12×10^{-3}	Estimated
$d_{\rho,0}$ $[mm^2/hr]$	Baseline Fibroblast Diffusivity	6.12×10^{-5}	[4]
p_ρ $[1/hr]$	Fibroblast Proliferation	9×10^{-4}	Estimated
$K_{\rho,c}$ [-]	Proliferation Saturation due to Cytokine	1×10^{-5}	[5]
$p_{\rho,e}$ $[1/hr]$	Mechanoregulation of Fibroblast Proliferation	$p_\rho/2$	[6]
$K_{\rho,\rho}$ [-]	Fibroblast Division Saturation	$550, 512.6$	[7]
d_ρ $[1/hr]$	Fibroblast Death Rate	$p_\rho(1 - \rho_{phys}/K_{\rho\rho})$	[7]
$p_{c,\rho}$ $[1/hr]$	Fibroblast Secretion of Cytokine	1.635×10^{-18}	[5]
$p_{c,e}$ $[1/hr]$	Mechanoregulation of Cytokine	5.45×10^{-18}	[5]
$K_{c,c}$ $[mol/mm^3]$	Cytokine Saturation	1	[5]
d_c $[1/hr]$	Cytokine Death Rate	0.005	Estimated
ρ_0 $[cells/mm^3]$	Nominal Fibroblast Density	55051	Estimated
c_0 $[g/mm^3]$	Initial Cytokine Concentration Inside Cavity	1×10^{-4}	[5]

Table 1: Parameters for the biochemical model. Parameters listed as estimated were selected in this work or modified from our previous wound healing models [5, 6].

Parameter	Description	Value	Reference
k_0 $[MPa]$	Linear Stiffness	6.375×10^{-3}	Estimated
k_1 $[MPa]$	Compressibility	0.317	Estimated
k_f $[MPa]$	Fiber Stiffness	0.015	[8]
k_2 [-]	Nonlinear Stiffening	0.048	[8]
γ_e [-]	Shape of Mechanosensing Curve	5	[5]
ϑ_e [-]	Midpoint of Mechanosensing Curve	2	[5, 9]
$K_{t,c}$ $[-]$	Traction Saturation due to Cytokine	1×10^{-5}	[5]
$K_{\phi,c}$ $[-]$	Collagen Production Saturation due to Cytokine	1×10^{-4}	[5]
p_{ϕ_e} $[1/hr]$	Collagen Production Activated by Stretch	p_ϕ	[5]
$K_{\phi,\rho}$ $[-]$	Collagen Production Saturation due to Collagen Fraction	$(\rho_0 * p_\phi)/d_\phi - 1$	[5]
d_ϕ $[1/hr]$	Collagen Degradation	9.7×10^{-4}	[10]
$d_{\phi,c}$ $[1/hr]$	Collagen Degradation Activated by Cytokine	8.81×10^{-5}	[10]
τ_ω $[hr]$	Time Constant for Reorientation	$10/(K_{\phi,\rho} + 1)$	[5]
τ_κ $[hr]$	Time Constant for Dispersion	$1/(K_{\phi,\rho} + 1)$	[5]
γ_κ [-]	Shape of Dispersion Rate Curve	2	[5]

Table 2: Parameters for the fully coupled mechanobiological model. Parameters listed as estimated were selected in this work or modified from our previous wound healing model [5, 6].

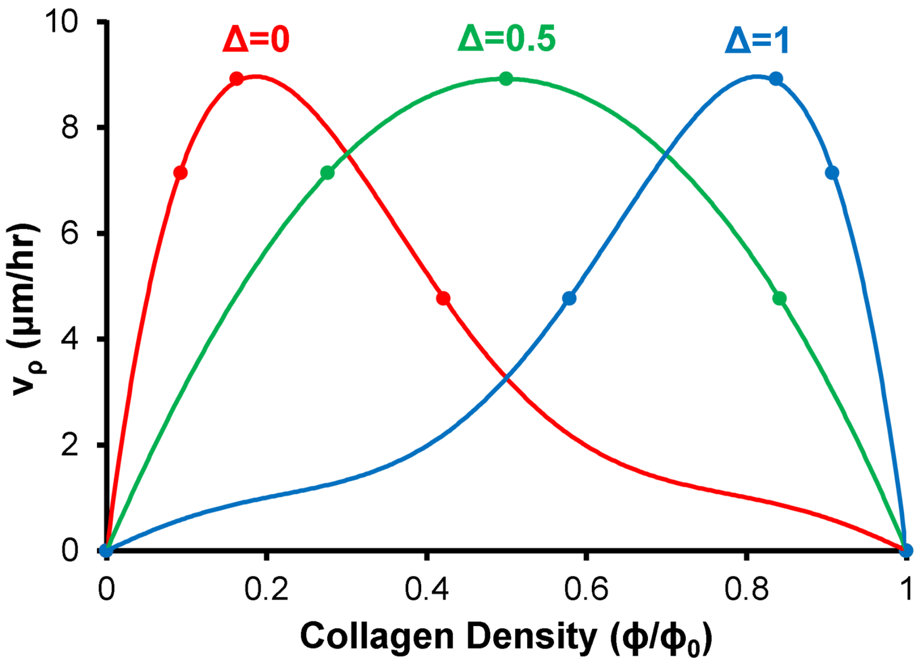

Figure 1: Fibroblast speed with respect to collagen density ($v_\rho(\phi)$) and its dependency on Δ. Example $v_\rho(\phi)$ curves are shown with Δ= 0 (red), 0.5 (green), 1 (blue). The function $v_\rho(\phi)$ was initially informed through [11, 12] through 5 data points displayed on the line $\Delta = 0$ while assuming $v_\rho(\phi) = 0$ for ϕ= 0 and 1. Due to the limited data and uncertainty, parameter Δ was created to shift the 5 data points and skew the interpolated function. Δ was further investigated in the biochemical GP, where it was determined that the optimum value was $\Delta = 0$.

Histological Image Analysis Methodology

Quantifying Fibroblast Density

1. Count Red Blood Cells (RBC)
   (a) Adjust Color Balance ([Minimum, Maximum])
      i. Red: [0,0]
      ii. Green: [0,100]
      iii. Blue: [0,0]
   (b) Convert Image Type From RGB Color to 32-bit
   (c) Apply Threshold ([0,$\sim$ 220])
   (d) Apply Watershed Segmentation
   (e) Analyze Particles for RBC Count
      i. Cell Size ([Minimum, Maximum]): [4,∞]
2. Count All Cells
   (a) Adjust Color Balance ([Minimum, Maximum])
      i. Red: [70,220]
      ii. Green: [0,0]
      iii. Blue: [0,0]
   (b) Convert Image Type From RGB Color to 32-bit
   (c) Apply Threshold ([0,$\sim$ 245])
   (d) Apply Watershed Segmentation
   (e) Analyze Particles for All Cell Count
      i. Under "Set Measurements" Select Original Slide Under "Redirect to:"
      ii. Cell Size ([Minimum, Maximum]): [4,∞]
   (f) Evaluate Modal Gray Value ($\leq\sim$125) to Isolate and Count Immune Cells
3. Fibroblast Count = All Cell Count - RBC Count - Immune Cell Count

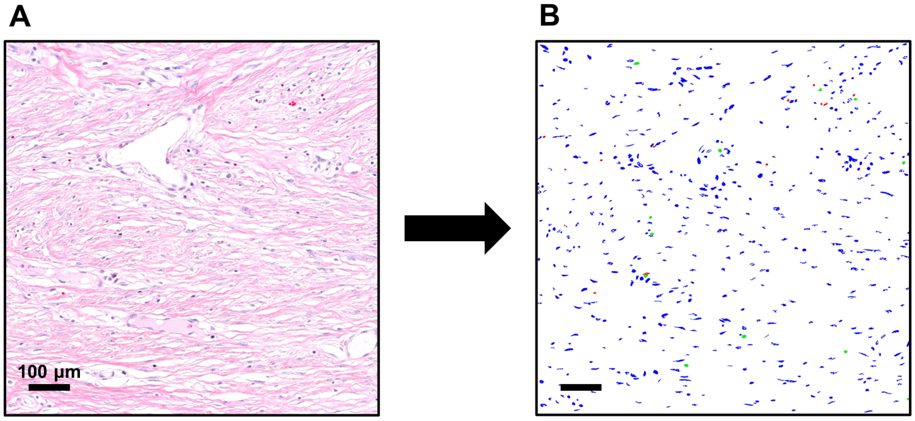

Figure 2: Result of post-processing individual regions (500 x 500 μm^2) obtained from porcine lumpectomy histology slides. (A) Regions were captured through Aperio ImageScope sampling across the entire cavity domain. Pictured is an example region from a histology slide 16 weeks post-surgery. (B) Using the procedures described above, regions were processed in ImageJ to quantify the number of fibroblasts (blue), red blood cells (red), and immune cells (green).

Calculating Collagen Density

1. In the 500 x 500 μm^2 histology region, select a small rectangular area (~100 μm^2) that contains no cells.

2. Measure for average pixel intensity in the small rectangular area. Note: Pixel intensity varies between 0 (black) and 255 (white).

3. Repeat steps 1 and 2 for a 500 x 500 μm^2 histology region that contains healthy breast connective tissue.

4. Calculate the estimated collagen density through the following equation:

$$(\phi/\phi_0)_{est.} = \frac{I_{scar} - 255}{0.3 * (I_{connective} - 255)}$$

where I_{scar} is the intensity of the scar tissue at the analyzed week and $I_{connective}$ is the intensity of the connective tissue.

References

[1] B. D. Cumming, D. McElwain, Z. Upton, A mathematical model of wound healing and subsequent scarring, Journal of The Royal Society Interface 7 (42) (2010) 19–34.

[2] D. C. Koppenol, F. J. Vermolen, F. B. Niessen, P. P. van Zuijlen, K. Vuik, A mathematical model for the simulation of the formation and the subsequent regression of hypertrophic scar tissue after dermal wounding, Biomechanics and modeling in mechanobiology 16 (2017) 15–32.

[3] K. E. Murphy, C. L. Hall, P. K. Maini, S. W. McCue, D. S. McElwain, A fibrocontractive mechanochemical model of dermal wound closure incorporating realistic growth factor kinetics, Bulletin of mathematical biology 74 (2012) 1143–1170.

[4] L. Olsen, J. A. Sherratt, P. K. Maini, A mechanochemical model for adult dermal wound contraction and the permanence of the contracted tissue displacement profile, Journal of theoretical biology 177 (2) (1995) 113–128.

[5] A. B. Tepole, Computational systems mechanobiology of wound healing, Computer Methods in Applied Mechanics and Engineering 314 (2017) 46–70.

[6] D. O. Sohutskay, A. B. Tepole, S. L. Voytik-Harbin, Mechanobiological wound model for improved design and evaluation of collagen dermal replacement scaffolds, Acta biomaterialia 135 (2021) 368–382.

[7] C. Valero, E. Javierre, J. M. García-Aznar, M. J. Gómez-Benito, A cell-regulatory mechanism involving feedback between contraction and tissue formation guides wound healing progression, PloS one 9 (3) (2014) e92774.

[8] A. B. Tepole, H. Kabaria, K.-U. Bletzinger, E. Kuhl, Isogeometric kirchhoff–love shell formulations for biological membranes, Computer methods in applied mechanics and engineering 293 (2015) 328–347.

[9] A. B. Tepole, C. J. Ploch, J. Wong, A. K. Gosain, E. Kuhl, Growing skin: a computational model for skin expansion in reconstructive surgery, Journal of the Mechanics and Physics of Solids 59 (10) (2011) 2177–2190.

[10] G. Laurent, Dynamic state of collagen: pathways of collagen degradation in vivo and their possible role in regulation of collagen mass, American Journal of Physiology-Cell Physiology 252 (1) (1987) C1–C9.

[11] T. J. Puls, C. S. Fisher, A. Cox, J. M. Plantenga, E. L. McBride, J. L. Anderson, C. J. Goergen, M. Bible, T. Moller, S. L. Voytik-Harbin, Regenerative tissue filler for breast conserving surgery and other soft tissue restoration and reconstruction needs, Scientific Reports 11 (1) (2021) 2711.

[12] D. O. Sohutskay, T. J. Puls, S. L. Voytik-Harbin, Collagen self-assembly: biophysics and biosignaling for advanced tissue generation, Multi-scale Extracellular Matrix Mechanics and Mechanobiology (2020) 203–245.
